# Effects of probiotic *Lactobacillus paracasei* TD3 on moderation of cholesterol biosynthesis pathway in rats

**DOI:** 10.22038/ijbms.2019.33933.8073

**Published:** 2019-09

**Authors:** Abolfazl Dehkohneh, Parvaneh Jafari, Hossein Fahimi

**Affiliations:** 1 Department of Biotechnology, Faculty of Advanced Science and Technology, Tehran Medical Sciences, Islamic Azad University, Tehran, Iran; 2 Microbiology Department, Faculty of Science, Islamic Azad University, Arak Branch, Arak, Iran; 3 Department of Genetics, Faculty of Advanced Science and Technology, Tehran Medical Sciences, Islamic Azad University, Tehran, Iran

**Keywords:** 3-hydroxy-3-methylglutaryl-CoA Reductase (HMGCR), Cholesterol, Cholesterol 7α-hydroxylase - (CYP7A1), Lactobacillus paracasei, Probiotics, Rat

## Abstract

**Objective(s):**

Prevalence of high-fat food consumption, such as fast foods is one of the major causes of hypercholesterolemia, which can lead to cardiovascular diseases. 3-hydroxy-3-methylglutaryl-coenzyme A reductase (HMGCR) and cytochrome P450 7A1 (CYP7A1) are two key genes in cholesterol metabolism. Use of probiotics in the diet is a promising approach for modulation of serum lipid. To confirm the modulation of serum lipids by probiotics, in this study, we have examined the efficacy of *Lactobacillus paracasei* TD3 in improving blood cholesterol levels.

**Materials and Methods:**

21 male Wistar rats were divided into three groups randomly (n=7). G1: negative control with normal diet, G2: positive control with high-fat diet, G3T: test group with high-fat diet plus supplementation with *L. paracasei *TD3 (10^10 ^CFU). In the 21st day, the rats were anesthetized using chloroform and then sacrificed. Blood samples were collected to analyze lipid panel parameters and hepatic enzymes by the auto-analyzer system. Adipose tissue samples were analyzed using real-time PCR for HMGCR and CYP7A1 genes expression.

**Results:**

Consumption of *L. paracasei* TD3 could reduce serum cholesterol levels significantly (*P*<0.05); whereas, there was no significant difference between experimental groups for triglycerides, LDL, and HDL levels. Aspartate aminotransferase (AST) and Alanine aminotransferase (ALT) enzymes were significantly decreased in the probiotic group. Furthermore, expression of HMGCR and CYP7A1 genes was dramatically declined in the probiotic group. There was no significant change in either uric acid or urea between the control and treated groups.

**Conclusion:**

Introduction of *L. paracasei* TD3 in rat’s diet can modulate serum cholesterol levels.

## Introduction

Hypercholesterolemia has been recognized for a long period as a leading risk factor for coronary heart diseases (1, 2). The world health organization (WHO) has predicted that until 2030 cardiovascular disease will remain the major cause of death, affecting approximately 23.6 million people worldwide (3). Also, it has been reported that a 1% decline in total serum cholesterol can reduce the risk of cardiovascular disease by 2 to 3% (4). Drug treatment is a pioneering approach for disease management in which 3-hydroxy-3-methylglutaryl coenzyme A (HMG-CoA) inhibitors like statins have been used widely to inhibit cholesterol biosynthesis. HMG-CoA, which is synthesized from acetyl CoA and acetoacetyl CoA by HMG-CoA synthase, is an intermediate in the mevalonate and ketogenesis pathways (5). However, several studies indicated that although these drugs have convincing results in cholesterol lowering, they have undesirable side effects (6, 7). Therefore, general tendency among researchers has increased to find a novel alternative method.

Numerous studies have shown that probiotic strains have several beneficial effects, including anti-cancer effect, boosting the immune system, improving gastrointestinal tract, hypocholesterolemic effect, and modulating lipid metabolism (8-10). According to the WHO definition, probiotic refers to live microorganisms which, when administrated in sufficient amount confers health benefits on the host. Lactic acid bacteria (LAB) are the most common probiotics that produce lactic acid as a bioactive compound. Two major groups of LAB are *Lactobacillus* and *Bifidobacterium*. Some strains of *Lactobacillus* SPP and *Bifidobacterium* SPP have been selected and used widely in food (dietary) supplements and dairy industry. *Lactobacillus casei* is a common species that is commonly found in the human gastrointestinal tract. In this study, the effects of consumption of *Lactobacillus paracasei* TD3 (as a native Iranian probiotic) on serum lipid profile and expression level of HMGCR (3-hydroxy-3-methylglutaryl-coenzyme A reductase) and CYP7A1 (also known as 7α-hydroxylase) genes in male Wistar rats with a high-fat diet were investigated. 

## Materials and Methods

***Animal grouping and diets***

Twenty-one male Wistar rats (6–8 weeks old, weighing 100–150 g) were purchased from Baqiyat-Allah Research Center (Tehran-Iran) and adapted to the laboratory conditions (22–24 ^°^C, 50–60% relative humidity and 12:12 hr light/dark cycle), with free access to food and water for two weeks. After the acclimatization period, the rats were randomly allocated to three experimental groups (seven rats per group). Group 1 (G1) with normal diet plus 1 ml phosphate buffered saline (PBS); group 2 (G2) was fed cholesterol-enriched diet plus 1 ml PBS; Group 3 (G3T) was fed a cholesterol-enriched diet supplemented with *L. paracasei* TD3 (10^10 ^CFU/ml). PBS and probiotic suspension were administered by oral gavage once daily at a certain time. The ingredients of normal and high-fat diets are shown in Table 1. All procedures were performed in accordance with the guidelines of the Medical Ethics Committee of Tarbiat Modares University, Tehran, Iran.

***Blood sampling and biochemical analysis***

On the 21st day, after 12 hr starvation, the rats were anesthetized by chloroform and fasting blood samples were collected by cardiac puncture. Then the serum samples were analyzed for total cholesterol (TC), triglyceride (TG), high-density lipoprotein (HDL), low-density lipoprotein (LDL), Alanine aminotransferase (ALT), Aspartate aminotransferase (AST), Alkaline phosphatase (ALP), and fasting blood sugar (FBS). Biochemical analysis of serum was performed using an auto-analyzer (RA1000-USA).

***Analysis of gene expression***

After blood collection, the rats were dissected immediately, and adipose tissues (approximately 0.5 g) were excised from the abdominal area of each rat and stored at RNX-Plus solution (SinaClon, Iran) for further steps. Total RNA was isolated using RNX-plus reagent according to the manufacturer’s protocol (SinaClon, Iran) and total cDNA was synthesized from total RNA (1 µg) using cDNA reverse transcriptase kit (Bioneer, Korea) as the template for quantitative polymerase chain reaction (qPCR). Expression levels of HMGCR and CYP7A1 genes were investigated using SYBRGreen (Takara, Japan) and a real-time PCR system (ABI-Step one, USA). GAPDH and G6PD housekeeping genes were used for normalization; after evaluation of the threshold cycle (Ct) value of internal control genes, the gene GAPDH was selected. The program used for relative gene expression investigation was 5 min at 94 ^°^C for initial denaturation, followed by 45 cycles of 94 ^°^C for 15 sec (denaturation), 59 ^°^C for 30 sec (annealing) and 72 ^°^C for 30 sec (extension), and the latest stage was 72 ^°^C for 10 min as the final extension. Primers were designed by using Primer3plus software and then analyzed using OligoAnalyzer 3.1 (Table 2). The relative gene expression levels were calculated using the 2^-^^ΔΔCt^ formula. The entire assay was carried out in triplicate for each sample.

***Statistical analysis***

All data expressed as mean±SEM. The significant difference between experimental groups was determined using Student’s t-test method. All statistical tests were carried out using the Graph-pad Prism software. In the entire experiment, the *P-value* less than 0.05 means significant alteration in which *P*<0.05, *P*<0.01, and *P*<0.001 values are shown by A, B, and C, respectively. 

## Results

***Body weight and fasting blood sugar***

The G2 group showed the highest level of body weight gain among experimental groups. FBS levels of G2 and G3T groups were elevated 18.8% and 25.4%, respectively; although they were not statistically significant.

***Total cholesterol***

**Figure 1:**
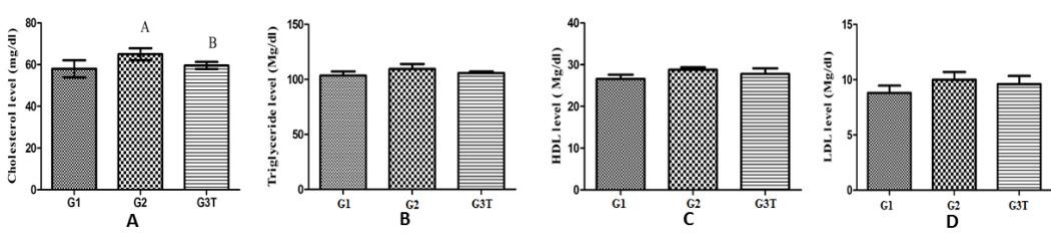
A) Mean concentration of total cholesterol in control and probiotic groups: Group G2 had the highest level among groups due to having a high-fat diet while Group G3T was decreased significantly in comparison with the G2 group due to probiotics consumption; B) Mean concentration of serum triglycerides in control and probiotic groups: There were no significant changes in experimental groups. Although G2 and G3T had different diets, it did not affect the triglyceride levels; C) Mean concentration of HDL in control and probiotic groups: There was no significant difference between the three experimental groups. Thus, the HDL level seems not to be affected by the high-fat diet and consumption of probiotics; D) Mean concentration of LDL in control and probiotic groups: Due to the high-fat diet, the G2 group showed the highest level. Consumption of probiotic decreased the LDL level in G3T

Total cholesterol level was significantly increased by 12% in the G2 group and decreased by 9.2% in the probiotic group (*P*<0.05) (Figure 1A). 

***Triglycerides***

There were no significant changes in group serum triglyceride levels. Also, the rats with a high-cholesterol diet displayed the highest level of TG (Figure 1B).

**Figure 2:**
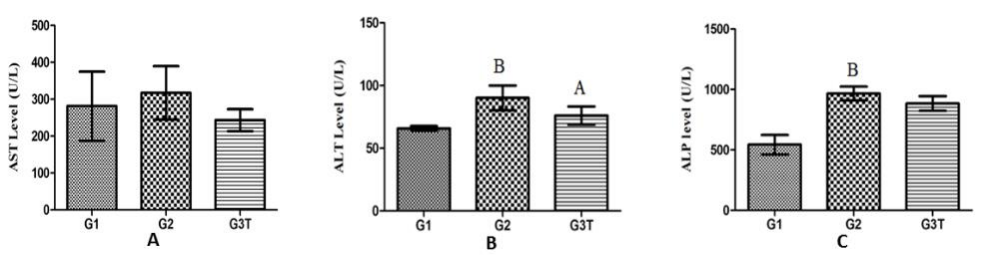
A) Serum AST levels in control and probiotic groups: Consumption of high-fat food led to elevation of AST level in the G2 group, while it decreased significantly in the G3T group; B) Serum ALT levels in control and probiotic groups: The ALT levels in G2 and G3T groups were increased because of the high-fat diet. However, L. paracasei TD3 caused significant reduction in the ALT level; C) Serum ALP levels in control and probiotic groups: Although the high-fat diet caused ALP elevation in G2 and G3T, there was no significant decrease in G3T group

**Figure 3:**
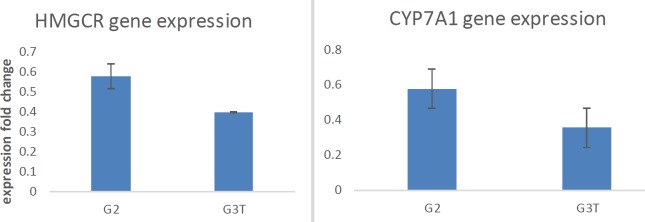
Gene expression analysis. The expression of both HMGCR and CYP7A1 genes were significantly decreased in the probiotic-treated group

**Table1:** Normal and high-fat diet ingredients. The high-fat diet includes all of the normal diet ingredients but the amount of fat is elevated by 20%.

**Material**	**Amount (%)**
Protein	23
Salt	0.55
Lysine	1.15
Methionine	0.33
Threonine	0.72
Tryptophan	0.25
Calcium	1
fat (in high-fat diet)	8

**Table 2:** List of primers used in this study

**Gene**	**primer sequence (5'** **-3'** **)**	**PCR product size (bp)**
HMGCR	F: AAGCTGTCATTCCAGCCAAG R: GGCCACATGCAATGTAGATG	171
CYP7A1	F: CTGCGTGACGAAATTGACAG R: CTTGCACTTCACGGATGATG	169
GAPDH	F: TTGTGATGGGTGTGAACCAC R: AGTCTTCTGAGTGGCAGTGATG	170
G6PD	F: CTTCCACCAAGCTGATACACAC R: TCACTCTGTTTGCGGATGTC	180

**Table 3:** The level of lipid panel parameters. Cholesterol level in G3T group was significantly decreased due to the administration of Lactobacillus paracasei TD3. Other lipid panel parameters had no significant change

**Groups**	**Control** ^–^ ** (G1)**	**Control** ^+^ ** (G2)**	**Probiotic (G3T)**	***P-value***
**Lipid profile**				
**Total cholesterol**	58.00 ± 1.87	65.00 ± 1.3	59.60 ± 0.74	0.007 ^(B)^
**Triglyceride**	103.4 ± 3.86	109.4 ± 4.67	105.8 ± 1.46	0.483
**HDL**	26.60 ± 1.03	28.80 ± 0.58	27.80 ± 1.35	0.5173
**LDL**	8.800 ± 0.66	10.00 ± 0.7	9.600 ± 0.74	0.70
**LDL/TC ratio**	0.1460 ± 0.006	0.1640 ± 0.005	0.1520 ± 0.004	0.1281
**HDL/TC ratio**	0.4520 ± 0.005	0.4380 ± 0.015	0.4640 ± 0.028	0.4455
**HDL/TG ratio**	0.2540 ± 0.015	0.2620 ± 0.01	0.2580 ± 0.013	0.8226

**Table 4:** The levels of Hepatic enzymes, uric acid, and urea in control and probiotic groups. The G2 group had the highest level and the biggest changes in all items. There was a significant decrease in AST and ALT levels in the G3T group. Also, the levels of uric acid and urea in G2 and G3T groups were already the same

** Groups**	**G1**	**G2**	**G3T**	***P-value***
**Hepatic enzymes**				
**AST**	281.4 ± 41.83	317.2 ± 32.46	243.4 ± 13.27	0.0684
**ALT**	65.80 ± 0.8602	90.20 ± 4.398 ^(A)^	76.00 ± 3.271 ^(A)^	0.0321
**ALP**	544.2 ± 80.70	967.2 ± 57.73 ^(B)^	884.6 ± 60.41	0.3518
**uric acid**	2.520 ± 0.2396	2.960 ± 0.2542	2.220 ± 0.2888	0.0906
**Urea**	48.80 ± 4.164	55.60 ± 3.027	55.60 ± 1.965	1.0

***High-density cholesterol (HDL)***

Measurement of HDL level in serum samples of different groups revealed no significant change (Figure 1C).

***Low-density cholesterol (LDL)***

The comparisons between tested groups demonstrated that the level of serum LDL in the rats with a fat-enriched diet (G2 group) was elevated significantly (Figure 1D). The reduction in LDL level in G3T revealed that *L*. para*casei* TD3 could decrease LDL, although it was not significant. Results of analysis of the serum lipid panel, including cholesterol, TG, HDL, and LDL are summarized in Table 3.

***Hepatic enzymes analysis***

Analysis of hepatic enzymes in three different groups revealed that aspartate aminotransferase (AST) (Figure 2A) and alanine aminotransferase (ALT) (Figure 2B) in the probiotic group were significantly decreased in G3T group in comparison with high-fat diet group (G2). However, the level of alkaline phosphatase had a slight reduction in the probiotic group (Figure 2C and Table 4).

***Uric acid and urea levels***

The results of serum analysis demonstrated that there was no significant change in either uric acid or urea in G2 and G3T groups (Table 4).

***Gene expression analysis***

In order to study the moderator effect of *L. paracasei* TD3 on the lipid profile, the rate of expression of two critical genes (HMGCR and CYP7A1) in the biosynthetic pathway of cholesterol was studied. The results from real-time PCR assay revealed that the both HMGCR and CYP7A1 genes expression were dramatically decreased in the probiotic group in comparison with the positive group (Figure 3).

## Discussion

Previous studies indicated that some strains of probiotics could have beneficial effects on the lipid profiles of blood. Some *lactobacillus* strains, including *L. casei, L. rhamnosus, *and* L. acidophilus, *have cholesterol and triglyceride lowering properties (11-14). Also, recent findings have shown that probiotics can moderate lipid metabolism by altering the expression of genes that are involved in lipid biosynthesis pathway (15, 16). Previously reported clinical trials have demonstrated the significant effect of probiotic and synbiotic supplementation in reduction and moderation of triglycerides, VLDL-cholesterol, and total cholesterol concentration. However, they indicated that probiotics did not have a significant effect on the whole of the blood lipid profile (17, 18). These *in vitro* and *in vivo* studies have suggested several cholesterol-lowering mechanisms. There are several accepted mechanisms of the hypocholesterolemic effect of bacteria in the small intestine, which include promotion of cholesterol binding, inhibition of reabsorption of bile acid by probiotic bacteria, and cholesterol attachment to probiotics’ cell-walls (19-21)

This study has focused on the effect of *L. paracasei* TD3 on lipid panel parameters. The results indicated that the consumption of *L. paracasei* TD3 has the potential to decrease cholesterol and triglycerides by 8 and 3%, respectively. Indeed, analysis of lipid profile and gene expression indicated that there was high adiposity in the high-fat diet group (G2 group) compared to the probiotic group. On the other hand, the G2 group had the highest level of total cholesterol and triglycerides among experimental groups. Although in general, the total cholesterol and triglycerides were decreased, only the decrease in total cholesterol was significant. The observed reduction in the total cholesterol in the probiotic group is in agreement with previous studies (22, 23). Another clinical trial study also showed that consumption of probiotic supplements in patients with gestational diabetes mellitus has beneficial effects on glycaemic control, triglycerides, and VLDL cholesterol concentrations (24). In agreement to several previous studies, the expression levels of the HMGCR gene and in contrast to some results, the expression level of the CYP7A1 gene was dramatically decreased in rats of the probiotic group (25-28). It is probable that *L. paracasei* TD3 bacteria release bioactive compounds that act as inhibitors for HMGCR gene expression. 

In a study, Grunewald reported that consumption of fermented milk leads to the meaningful reduction of total cholesterol in serum (29). Also, it has been reported that consumption of yogurt containing *Bifidobacterium pseudocatenulatum *G4 or *Bifidobacterium longum *BB536 can reduce total cholesterol, LDL, and VLDL levels in serum (30). Moreover, another study indicated that using probiotic *Lactobacillus*, especially *L. reuteri* and *L. plantarm*, could reduce total cholesterol and LDL-Cholesterol. They also have beneficial effects on triglycerides and HDL-cholesterol (31). Recently, Yadav *et al*. examined the cholesterol-lowering potential of *L. fermentum* MTCC: 5898 in rats with a high-fat diet. The results of this study indicated that consumption of *L. fermentum* could attenuate total cholesterol, triglycerides, VLDL, and LDL; while a high level of HDL was observed in the probiotic-treated group (32). In another similar study, Aminlari *et al*. evaluated the effect of two probiotics bacteria, *L. plantarum*, and *Bacillus coagulans,* on lipid panel parameters. They observed that probiotics groups had decreased serum concentrations of total cholesterol, triglycerides, LDL, and VLDL in comparison with the enriched-cholesterol diet group. However, the probiotics group had a higher level of HDL. Moreover, the results demonstrated that ALT and AST were decreased upon consumption of probiotics (33). In agreement with the results of the present study, researchers illustrated that *L. plantarum* DR7 could decrease HMGCR gene expression (34). 

## Conclusion

Results revealed that consumption of *L. paracasei *TD3 for 21 days caused down-regulation of HMGCR and CYP7A1 genes. Furthermore, levels of AST and ALT hepatic enzymes (as two important indicators in the fatty liver) were significantly decreased. Administration of *L. paracasei* TD3 strain may prevent accumulation of fats in the liver and may have inhibitory effects on adiposis. 
